# Editorial: Noninvasive brain stimulation: a promising approach to study and improve emotion regulation

**DOI:** 10.3389/fnbeh.2025.1633936

**Published:** 2025-06-23

**Authors:** Masaru Tanaka, Zhenhong He, Shangfeng Han, Simone Battaglia

**Affiliations:** ^1^Danube Neuroscience Research Laboratory, HUN-REN-SZTE Neuroscience Research Group, Hungarian Research Network, University of Szeged (HUN-REN-SZTE), Szeged, Hungary; ^2^School of Psychology, Shenzhen University, Shenzhen, China; ^3^Division of Neuroscience and Experimental Psychology, School of Biological Science, University of Manchester, Manchester, United Kingdom; ^4^Department of Psychology and Center for Brain and Cognitive Sciences, School of Education, Guangzhou University, Guangzhou, China; ^5^Center for Studies and Research in Cognitive Neuroscience, Department of Psychology “Renzo Canestrari, ” Alma Mater Studiorum, University of Bologna, Bologna, Italy; ^6^Department of Psychology, University of Turin, Turin, Italy

**Keywords:** emotion regulation, transcranial magnetic stimulation, transcranial direct current stimulation, prefrontal cortex, kynurenine, precision medicine, neuromodulation, mental disorders

## 1 Introduction

Emotion regulation (ER) profoundly shapes our mental health and overall wellbeing, influencing daily decisions and social interactions (Kraiss et al., 2020; Cludius et al., 2020; Sloan et al., 2017; Chervonsky and Hunt, 2019; Hu et al., 2014; Berking and Wupperman, 2012). Effective ER strategies consistently correlate with better psychological and physical health outcomes, heightened life satisfaction, and improved resilience in the face of everyday stressors (Kraiss et al., 2020; Tamir et al., 2024; Raugh et al., 2025; Espenes et al., 2024; Schäfer et al., 2017; Troy et al., 2018). Conversely, insufficient or maladaptive ER can exacerbate mental health issues, impacting emotional balance and daily functioning, particularly in vulnerable populations like individuals with psychiatric disorders (Kraiss et al., 2020; Schäfer et al., 2017; Cludius et al., 2020; Tsujimoto et al., 2024; Lincoln et al., 2022; Polizzi and Lynn, 2021). Recent advances in noninvasive brain stimulation (NIBS), such as transcranial magnetic stimulation (TMS) and transcranial direct current stimulation (tDCS), offer powerful tools to modulate neural activity, particularly within the prefrontal cortex (PFC) (Regenold et al., 2022; Chan et al., 2021; Xiao et al., 2024; Pettorruso et al., 2021; Liu and Yuan, 2021; Brevet-Aeby et al., 2016; Sagliano et al., 2019). These techniques influence resting-state connectivity, neurotransmitter systems, and stress-related pathways, including cortisol regulation via the hypothalamic-pituitary-adrenal axis, which plays a critical role in disorders such as PTSD (Battaglia et al., 2025a; Tortora et al., 2023; Schuurmans et al., 2021; Jensen et al., 2024; Almeida et al., 2021; Lawrence and Scofield, 2024). Complementing circuit-based neuromodulation, plant-derived phytochemicals have emerged as multimodal agents capable of simultaneously targeting neuroinflammation, oxidative stress, and mitochondrial dysfunction in major depressive disorder (Figueiredo Godoy et al., 2025; Cordeiro et al., 2022; Picheta et al., 2024; Kabra et al., 2022; Mokhtari, 2022). Additionally, CRISPR/Cas9 evidence indicates that loss of kynurenine aminotransferase activity markedly disrupts cerebral mitochondrial respiration and ATP production, underscoring dysregulated tryptophan–kynurenine metabolism as a key bioenergetic contributor to emotion-related disorders (Juhász et al., 2025; Javelle et al., 2021; Marx et al., 2021; Tanaka et al., 2022; Mor et al., 2021; Skorobogatov et al., 2021; Szabó et al., 2025). Structure-guided tuning of this endogenous metabolite hints at next-generation neurotherapeutics (Tanaka et al., 2025). By influencing resting-state connectivity, neurotransmitter function, and deeper brain circuits, NIBS techniques are increasingly recognized for their therapeutic potential across psychiatric disorders, including depression, impulsivity, PTSD, insomnia, schizophrenia, and even developmental conditions like dyslexia (Zhou et al., 2024; Krystal et al., 2020; Wei et al., 2020; Konicar et al., 2022; Dunlop et al., 2017; Sathappan et al., 2019).

Current research gaps involve uncertainties regarding optimal and individualized brain stimulation targets for effectively modulating emotion regulation (Zhang et al., 2022; Shen and Zhou, 2024; Smits et al., 2020; Jahangard et al., 2019; Ironside et al., 2016; Salehinejad et al., 2017). Precise anatomical and functional connectivity between superficial cortical sites—especially within the prefrontal cortex—and deeper emotional brain circuits remains insufficiently defined (Sun et al., 2023; Berboth and Morawetz, 2021; Ben Shalom and Skandalakis, 2025; Skandalakis et al., 2023; Huang et al., 2020; Khastkhodaei et al., 2021). Additionally, there is limited understanding of how variations in brain connectivity profiles influence individual responses to stimulation including the interplay between central and autonomic nervous system markers such as cardiac deceleration during cognitive control processes (Di Gregorio et al., 2024; Ozdemir et al., 2021; A Papasavvas et al., 2022; Wang et al., 2021; Giannakakis et al., 2020; Marzetti et al., 2024). Moreover, standardized methods integrating imaging-based modeling, automated optimization techniques, and systematic group-level analyses to refine target selection and stimulation parameters are lacking, hindering personalized treatments and limiting our ability to predict therapeutic outcomes across different mental health conditions (Malekmohammadi et al., 2022; Treu et al., 2020; Tervo et al., 2020; Chen et al., 2021). This editorial synthesizes how the four contributions in this Research Topic advance non-invasive brain-stimulation science by mapping cortical–subcortical connectivity, testing innovative stimulation modalities, and demonstrating behavioral benefits, thereby narrowing gaps in target selection, mechanistic understanding, and individualized intervention strategies for emotion-regulation enhancement while outlining future research priorities and translational opportunities.

## 2 Articles

Using a within-subjects crossover design, Wang et al. compared a single 30-min bout of Tai Chi, isocaloric cycling, and quiet rest in 36 young adults while tracking emotional-memory performance and prefrontal hemodynamics with fNIRS. Tai Chi selectively boosted recall accuracy for positive images and produced a robust rise in oxy-hemoglobin within the left dorsolateral prefrontal cortex—a hub for cognitive reappraisal—whereas cycling yielded only modest gains and rest produced none. Critically, increases in left-DLPFC oxygenation strongly predicted memory accuracy, evidencing an exercise-specific pathway linking mind-body practice to enhanced emotion regulation circuitry. The findings position acute Tai Chi as a fast-acting, non-pharmacological modulator of prefrontal control over affective processing, illustrating how exercise modality shapes ER-related brain–behavior dynamics. These data encourage integrating mindful movement sessions into rehabilitation and mental-health programs targeting affect control.

Hou et al. deliver a convincing demonstration that precisely targeting a deeper cortical node—the left anterior cingulate cortex (ACC)—can restore social emotion-regulation (ER) functions. In FMR1-knock-out and valproic-acid mouse models of autism, socially triggered ultrasonic vocalizations (USVs), normally reliant on ER circuitry, fail to recruit ACC excitatory neurons. Optogenetic silencing of the same neurons suppresses USVs in wild-type animals, confirming necessity. Conversely, optogenetic activation or seven-day, millimeter-focused 10 Hz repetitive TMS—a novel three-coil “micro-TMS” capable of confining magnetic fields to ~0.5 mm3—reactivates left-ACC responsiveness, boosting call rates, syllable repertoire, and affective reciprocity in both mutant lines. Right-hemisphere or off-target stimulation leaves behavior unchanged, underscoring lateralized specificity. Importantly, gains persist for at least 1-week post-stimulation, hinting at durable circuit re-entrainment. The study positions the left ACC as a causal, therapeutically tractable hub linking cognition, affect, and social communication, charting a roadmap for human NIBS protocols targeting deeper cortical–subcortical ER networks.

Tomita et al. pioneer transcranial static magnetic field stimulation (tSMS) as a compact, low-cost tool for dampening maladaptive emotion-regulation processes in social anxiety. Using a triple-magnet device capable of suppressing cortical excitability up to 8 cm deep, they targeted the right frontopolar area—a hub where pathological self-focused attention (SFA) originates. In twenty-three high-SAD students, a single 20-min tSMS session reduced resting-state oxy-hemoglobin in right frontopolar channels, attenuated SFA toward bodily sensations, and increased adaptive field and detached-mindfulness perspectives during a subsequent impromptu speech. Benefits scaled with trait anxiety, suggesting precision engagement of overactive circuitry. Sham stimulation produced no comparable change. Critically, the protocol required no electrical current or acoustic coil discharge, minimizing discomfort and simplifying translation. By demonstrating that static magnetic fields can recalibrate attentional stance and relieve anxiety-linked ER distortions, the study opens a fresh avenue for future clinical deployment worldwide.

Moro et al. provide decisive evidence that the orbitofrontal cortex (OFC) is a causal node for impulsive inter-temporal choice. Forty-two healthy adults received a 20-min, 2 mA bilateral tDCS session with either anodal-left/cathodal-right or the reversed montage while completing a monetary delay-discounting task and an n-back working-memory test. Both polarities significantly increased area-under-the-curve values and lowered discounting rates, revealing steeper valuation of delayed rewards—effectively curbing impulsive decisions. Critically, stimulation left baseline trait impulsivity, Go/No-Go behavioral inhibition, and working-memory accuracy unchanged, ruling out non-specific executive confounds. Computational electric-field modeling confirmed maximal current density within medial–lateral OFC, isolating the target. The study clarifies that modulating OFC excitability alone recalibrates cost-benefit calculations without altering other cognitive domains, positioning OFC-focused tDCS as a promising intervention for impulsivity-driven disorders such as addiction and binge eating.

## 3 Conclusion and future directions

Taken together, the contributions assembled here narrow critical gaps in emotion-regulation science by elucidating neural circuits, establishing precision NIBS targets, and spotlighting pathways toward clinically translatable therapeutics. Specifically, the evidence shows that mindful exercise sharpens prefrontal ER networks, millimeter-targeted ACC stimulation revives social communication, portable static magnets mitigate social anxiety, and, orbitofrontal tDCS reins in impulsive choice. Future directions should prioritize personalized NIBS protocols informed by individual neural connectivity profiles, integration of NIBS with complementary neurotechnologies such as neurofeedback and advanced imaging, and systematic translation into clinical settings (Tanaka, 2024; Soleimani et al., 2023; Karimi et al., 2025; Guerrero Moreno et al., 2021; Raymond et al., 2022; Carè et al., 2024). These steps will accelerate effective, tailored interventions, substantially advancing mental health care—especially as we deepen our understanding of brain–body communication and confront the paradox of a brain studying itself, a pursuit that intertwines consciousness, self-regulation, and the boundaries of introspective neuroscience (Di Gregorio and Battaglia, 2024; Battaglia et al., 2025b; Tanaka, 2025; Signorelli et al., 2022; Northoff et al., 2020; Peters, 2025).
